# The structural and functional integrities of porcine myocardium are mostly preserved by cryopreservation

**DOI:** 10.1085/jgp.202313345

**Published:** 2023-07-03

**Authors:** Weikang Ma, Kyoung Hwan Lee, Christine E. Delligatti, M. Therese Davis, Yahan Zheng, Henry Gong, Jonathan A. Kirk, Roger Craig, Thomas Irving

**Affiliations:** 1 BioCAT, Department of Biology, Illinois Institute of Technology, Chicago, IL, USA; 2 Electron Microscopy Facility, UMass Chan Medical School, Worcester, MA, USA; 3 Department of Cell and Molecular Physiology, Loyola University Chicago, Chicago, IL, USA; 4 College of Basic Medical Sciences, Dalian Medical University, Dalian, China; 5 Division of Cell Biology and Imaging, Department of Radiology, University of Massachusetts Medical School, Worcester, MA, USA

## Abstract

Structural and functional studies of heart muscle are important to gain insights into the physiological bases of cardiac muscle contraction and the pathological bases of heart disease. While fresh muscle tissue works best for these kinds of studies, this is not always practical to obtain, especially for heart tissue from large animal models and humans. Conversely, tissue banks of frozen human hearts are available and could be a tremendous resource for translational research. It is not well understood, however, how liquid nitrogen freezing and cryostorage may impact the structural integrity of myocardium from large mammals. In this study, we directly compared the structural and functional integrity of never-frozen to previously frozen porcine myocardium to investigate the consequences of freezing and cryostorage. X-ray diffraction measurements from hydrated tissue under near-physiological conditions and electron microscope images from chemically fixed porcine myocardium showed that prior freezing has only minor effects on structural integrity of the muscle. Furthermore, mechanical studies similarly showed no significant differences in contractile capabilities of porcine myocardium with and without freezing and cryostorage. These results demonstrate that liquid nitrogen preservation is a practical approach for structural and functional studies of myocardium.

## Teaser

Our current comprehensive structural and functional study validates the use of liquid nitrogen–frozen and cryostored porcine myocardium as an excellent test bed for biophysical studies of cardiac muscle that may lead to more consistent experiment results requiring reduced numbers of animals.

## Introduction

Striated muscle is a multicellular hierarchical structured system that is responsible for force generation to power animal movement, maintain posture, and circulate the blood. The contractile unit, the sarcomere, is composed of interdigitating myosin-containing thick filaments and actin-containing thin filaments organized in a hexagonal lattice; interaction between the filaments converts the chemical energy from ATP into mechanical output as contractile force. Structural, biochemical, and mechanical experiments provide insights into the fundamental physiological mechanisms of muscle function and the underlying causes of myopathies.

While fresh muscle tissue works best for these studies, this is not always practical, especially for human tissues and for heart tissue from large animals. For rodents and other small-animal studies, it is more feasible to perform experiments using fresh muscle or freshly permeabilized tissue that may be stored in glycerol solution at −20°C for up to several weeks. However, even in this case, the short storage period of permeabilized muscle implies that additional animals may be required, with the attendant complications due to interanimal variation and the financial burden of obtaining the animals. For large animal studies, many researchers work on previously frozen muscle samples due to either high cost (e.g., porcine myocardium) or sample scarcity (human myocardium). Using previously frozen tissues allows researchers to schedule experiments more freely and preserve precious samples for further studies on the same batch of tissue.

Milburn et al. (2022) showed that freezing has minimal effects on contractile properties of human myocardium, and our preliminary studies showed that high-quality x-ray diffraction patterns can be obtained from previously frozen human (Ma et al., 2022a) and porcine myocardium under resting (Awinda et al., 2022; Jani et al., 2022; Ma et al., 2023) and contracting (Ma et al., 2022b) conditions. It was, however, not clear to what extent liquid nitrogen treatment and cryostorage may impact the structural integrity of myocardium from large mammals. This is a critical question to address, to ensure that the field is not led astray by artifacts that may be introduced from liquid nitrogen freezing and storage.

In this study, we compared the structural and functional integrity of never-frozen to previously frozen porcine myocardium to investigate the consequences of freezing and cryostorage. Our x-ray diffraction measurements from hydrated tissue under near-physiological conditions and electron microscopy (EM) images from chemically fixed, dehydrated porcine myocardium show that prior freezing has relatively minor effects on the structural integrity of the muscle. Our mechanical studies also show no significant differences in contractile capabilities of porcine myocardium with and without freezing and cryostorage. Taken together, our results from porcine myocardium show that liquid nitrogen preservation is a practical approach for long-term sample storage for structural and functional studies of permeabilized large mammalian myocardium.

## Materials and methods

### Muscle preparation for structural studies

Experiments used hearts (*n* = 2) from young (3–5 mo old) male Yucatan minipigs. Humane euthanasia and tissue collection procedures were approved by the Institutional Animal Care and Use Committees at Exemplar Genetics Inc. Hearts were washed and stored in cold del Nido cardioplegia solution (Nephron Pharmaceuticals Corporation) and shipped overnight to the BioCAT facility at the Advanced Photon Source, Argonne National Laboratory, Lemont, IL. Left ventricular muscle was permeabilized as described previously (Ma et al., 2021). Briefly, fresh muscle pieces (∼0.5 cm^3^) from left ventricular wall were cut from the hearts and permeabilized in skinning solution (pCa8: 2.25 mM Na_2_ATP, 3.56 mM MgCl_2_, 7 mM EGTA, 15 mM sodium phosphocreatine, 91.2 mM potassium propionate, 20 mM Imidazole, 0.165 mM CaCl_2_, 15 mM 2,3-butanedione 2-monoxime, creatine phosphate kinase 15 U/ml, 1% Triton X-100, and protease inhibitor cocktail [cOmplete Sigma-Aldrich]) for ∼30 min. Blunt-end forceps were used to split the muscle piece into smaller bundles and subsequently further split into 1-mm-diameter fiber bundles by peeling fibers from the ends of the tissue bundles. The fiber ends damaged by the forceps at the end of the preparations were then cut off. The 1-mm fiber bundles were transferred into fresh skinning solution either overnight at 4°C or at room temperature for 2–3 h on an agitator until the tissue became clear and transparent. Muscles were then washed with fresh relaxing solution (pCa8: 2.25 mM Na_2_ATP, 3.56 mM MgCl_2_, 7 mM EGTA, 15 mM sodium phosphocreatine, 91.2 mM potassium propionate, 20 mM imidazole, 0.165 mM CaCl_2_, creatine phosphate kinase 15 U/ml, and protease inhibitor cocktail) for ∼10 min, repeated three times to wash out the 2,3-butanedione 2-monoxime and Triton X-100. The muscles were further dissected into ∼200–300-μm-diameter fiber bundle strips, clipped with aluminum T-clips, and stored in cold (4°C) relaxing solution for the day’s experiments.

### Frozen muscle preparations

Pieces (∼0.5 cm^3^) of left ventricular wall muscle were dropped into a Dewar filled with liquid nitrogen for about 2 min until the muscle solidified. No cryo-protectants were used. The frozen muscle pieces were stored at −80°C until needed (1 mo for the current study). For experiments, the muscle pieces were first thawed by incubating in skinning solution and then permeabilized as described above for fresh tissue.

### Small-angle x-diffraction

X-ray diffraction patterns were collected from permeabilized fresh or frozen muscle strips using the small-angle instrument on the BioCAT beamline 18ID at the Advanced Photon Source, Argonne National Laboratory (Fischetti et al., 2004). The x-ray beam was focused to ∼0.06 × 0.15 mm at the detector plane. The sample-to-detector distance was ∼3 m and the x-ray wavelength was 0.103 nm. T-clipped fiber bundles were mounted between a force transducer (Model 402A; Aurora Scientific) and a static hook. Force was monitored using Muscle Dynamic Control system (Model 610A; Aurora Scientific). Sarcomere length was adjusted by laser diffraction using a 4 mW HeNe laser. Diffraction patterns were collected at a sarcomere length of 2.0 μm. X-ray exposures were 1 s at an incident flux of ∼3 × 10^12^ photons per second, and the patterns were collected on a CCD-based x-ray detector (Mar 165; Rayonix Inc). The data were analyzed using data reduction programs from the MuscleX software package developed at BioCAT (Jiratrakanvong et al., 2018). The equatorial reflections were measured by the “Equator” routine in MuscleX as described previously (Ma et al., 2018a). The intensities and spacings of meridional reflections were measured by the “Projection Traces” routine in MuscleX as described (Ma et al., 2018b). The intensities of the first myosin layer lines (I_MLL1_) were measured using the “Projection Traces” routine in MuscleX as described (Ma et al., 2021).

### Sample preparation for EM

Cardiac muscle fiber bundles from fresh or frozen (3–5 mo old) pig heart ventricular walls were permeabilized as described above and pinned down onto a Sylgard substrate under slight tension. Fibers were fixed with 2.5% glutaraldehyde in 0.1 M cacodylate buffer, pH 7.2, for 2 h at room temperature followed by continued fixation at 4°C overnight. Samples were then rinsed in cacodylate buffer three times for 20 min each at 4°C. The fibers were then shipped overnight on ice from Argonne National Laboratory to the University of Massachusetts Chan Medical School. The fibers were cut into small pieces (1 × 3 mm) and postfixed with 1% wt/vol OsO_4_ in distilled water, then dehydrated in a series of graded ethanol solutions, and embedded in Epon resin. Sections (70 nm thick) were stained with uranyl acetate followed by lead citrate (Reynolds, 1963) and examined in an FEI Tecnai G2 Spirit electron microscope at 120 kV. Images were collected on a 4 × 4 k CMOS camera (Gatan Rio 9). The A-band, Z-line, and sarcomere length were measured by the straight-line tool in ImageJ software (National Institutes of Health [NIH]). 20 sarcomeres were measured from each preparation and the numbers measured from each preparation were averaged.

### Sample preparation for mechanics measurements

Experiments used hearts (*n* = 3) from young (3–5 mo old) male Yucatan minipigs. Humane euthanasia and tissue collection procedures were approved by the Institutional Animal Care and Use Committee at Loyola University Chicago. Two adjacent pieces of tissue from porcine left ventricular apex were immediately isolated following excision of the heart. Skinned cardiomyocytes were isolated fresh from one piece, while the second piece was flash-frozen in liquid nitrogen and stored at −80°C for 2–4 wk before skinned myocytes were isolated. Skinned cardiomyocytes were prepared in a similar manner as previous papers (Martin et al., 2021). Briefly, tissue was placed in Isolation solution (5.8 mM Na_2_ATP, 7.1 mM MgCl_2_, 2 mM Ca^2+^-EGTA, 108 mM potassium chloride, 8.9 mM potassium hydroxide, 10 mM imidazole) containing protease and phosphatase inhibitors (1:100 ratio; Thermo Fisher Scientific) and 0.3% (vol/vol) Triton X-100. To control for the possibility of oxidation occurring during the freeze/thaw cycle, dithiothreitol was omitted. The tissue was mechanically homogenized in three 1-s bursts and left on ice for 20 min to permeabilize. Next, the myocyte solution was pelleted by centrifugation at 120 *g* for 2 min and resuspended in Isolation solution without Triton. To assess function, myocytes were first attached with UV-curing glue (NOA 61; Thorlabs) to two pins, one attached to a force transducer (Aurora Scientific) and the other to a high-speed piezo-length controller (Thorlabs). Next, the myocyte was exposed to a maximal calcium Activating solution (6.3 mM Na_2_ATP, 6.2 mM MgCl_2_, 10 mM Ca^2+^-EGTA, 10 mM sodium phosphocreatine, 28.1 mM potassium propionate, and 100 mM N,N-Bis(2-hydroxyethyl)-2-aminoethanesulfonic acid [48.6 µM free Ca^2+^]) to elicit contraction and then perfusion was switched to 100% Relaxing solution (6.2 mM Na_2_ATP, 6.5 mM MgCl_2_, 10 mM EGTA, 10 mM sodium phosphocreatine, 47.6 mM potassium propionate, and 100 mM N,N-Bis(2-hydroxyethyl)-2-aminoethanesulfonic acid) to facilitate relaxation. This process was continued with additional submaximal calcium solutions containing Activating solution mixed with Relaxing solution. All measurements were conducted at room temperature and performed at a sarcomere length of 2.1 µm as measured by Fast Fourier Transform.

### Statistics

**Figure 1.**
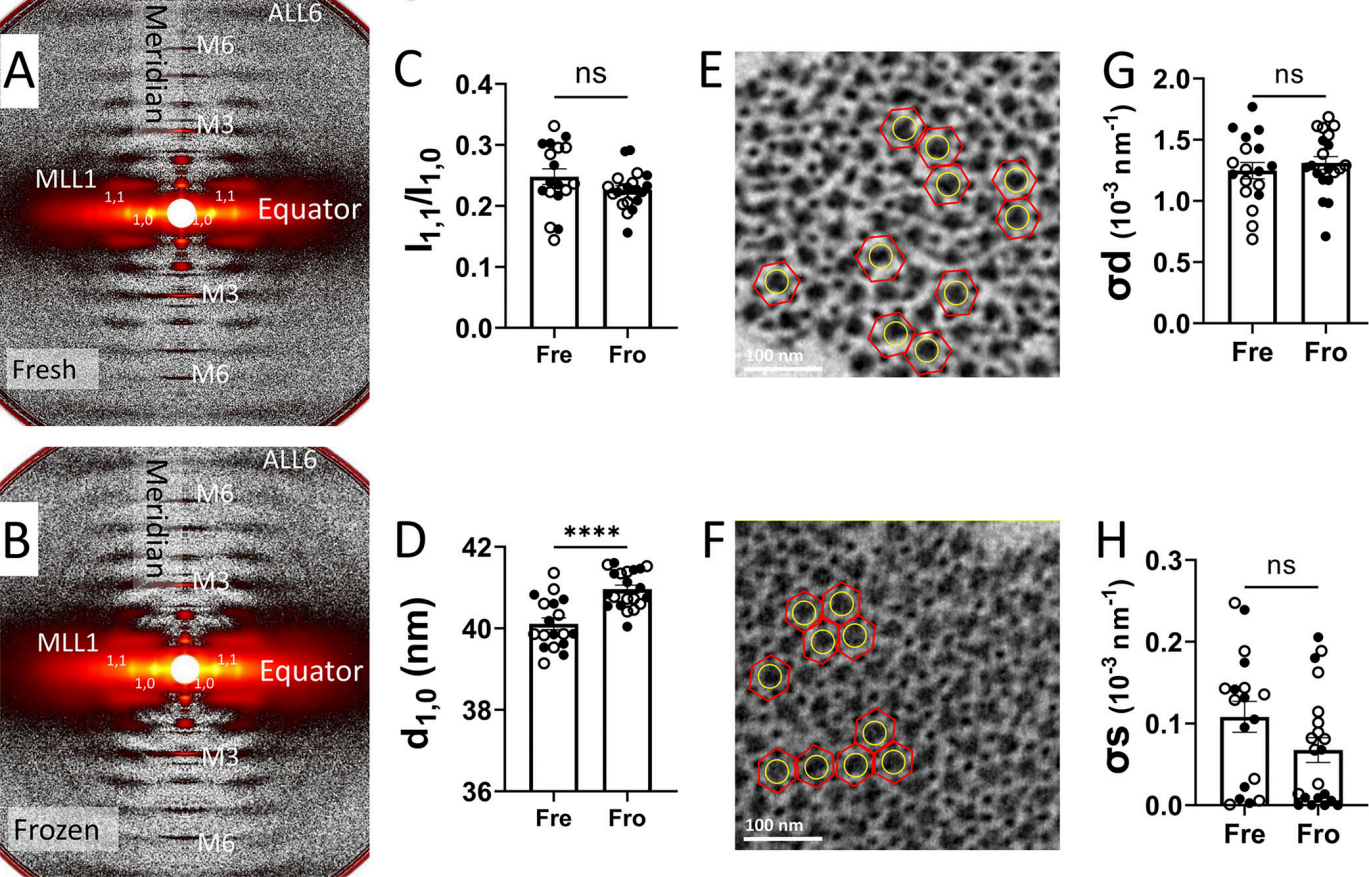
**Lattice structure of permeabilized fresh and frozen porcine myocardium. (A and B)** Representative x-ray diffraction patterns from permeabilized fresh (A) and frozen (B) porcine myocardium. **(C)** The equatorial intensity ratio (I_1,1_/I_1,0_) from permeabilized fresh (Fre) and frozen (Fro) porcine myocardium. **(D)** The lattice spacing (d_1,0_) from permeabilized fresh and frozen porcine myocardium. **(E and F)** The EM cross-sections are views of sarcomere A-bands from permeabilized fresh (E) and frozen (F) porcine myocardium. The thick filaments (yellow circles) are surrounded by six hexagonally arranged thin filaments (red hexagons). **(G and H)** σ_d_ (G) and σ_s_ (H) in fresh and frozen myocardium. ns: P *≥* 0.05, and ****: P < 0.0001. Solid and open symbols are from different biological repeats. The error bars in the graphs are SEM.

**Figure 2.**
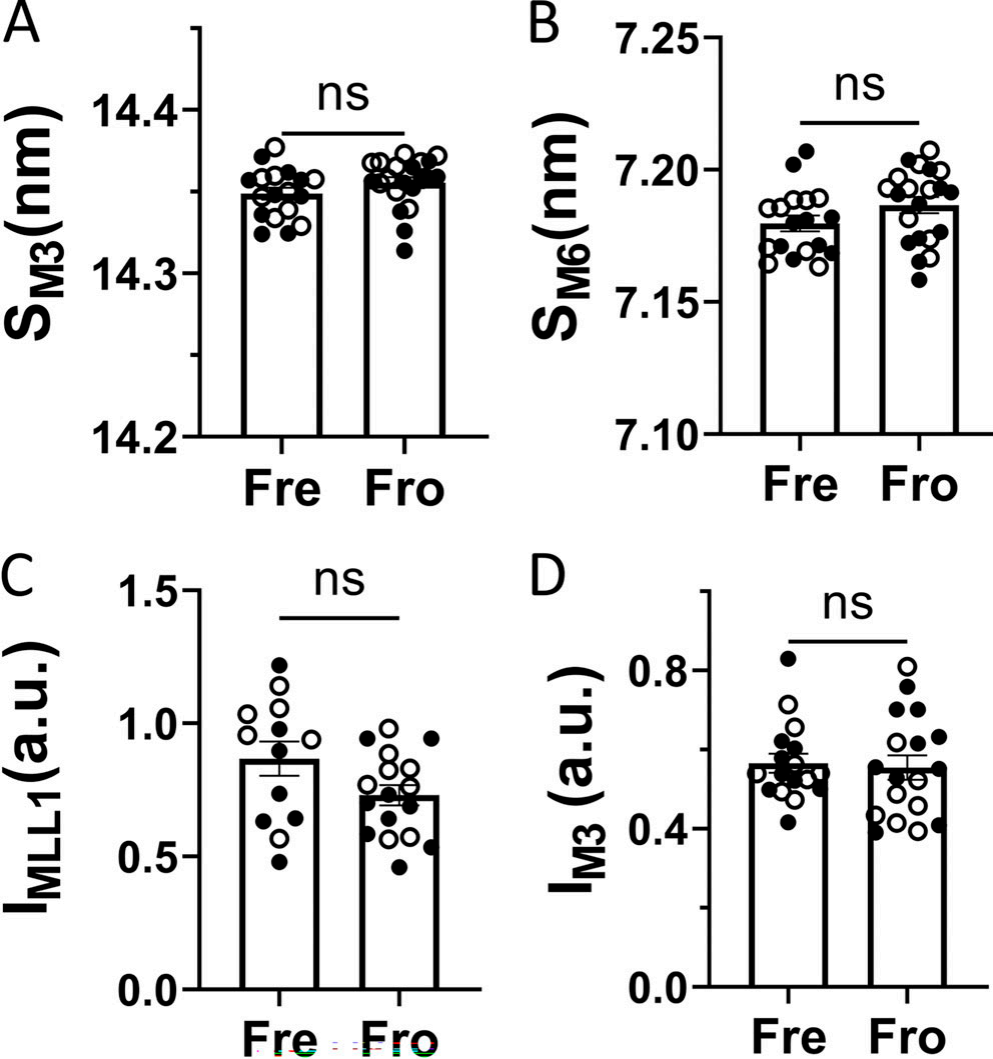
**X-ray diffraction properties of fresh and frozen porcine myocardium. (A and B)** The spacing of the third (A, S_M3_) and sixth (B, S_M6_) order meridional reflections from permeabilized fresh (Fre) and frozen (Fro) porcine myocardium. **(C)** I_MLL1_ from fresh and frozen porcine myocardium. **(D)** The intensity of the third-order meridional reflection (I_M3_) from fresh and frozen porcine myocardium. ns: P *≥* 0.05. Solid and open symbols are from different biological repeats. The error bars in the graphs are SEM.

**Figure 3.**
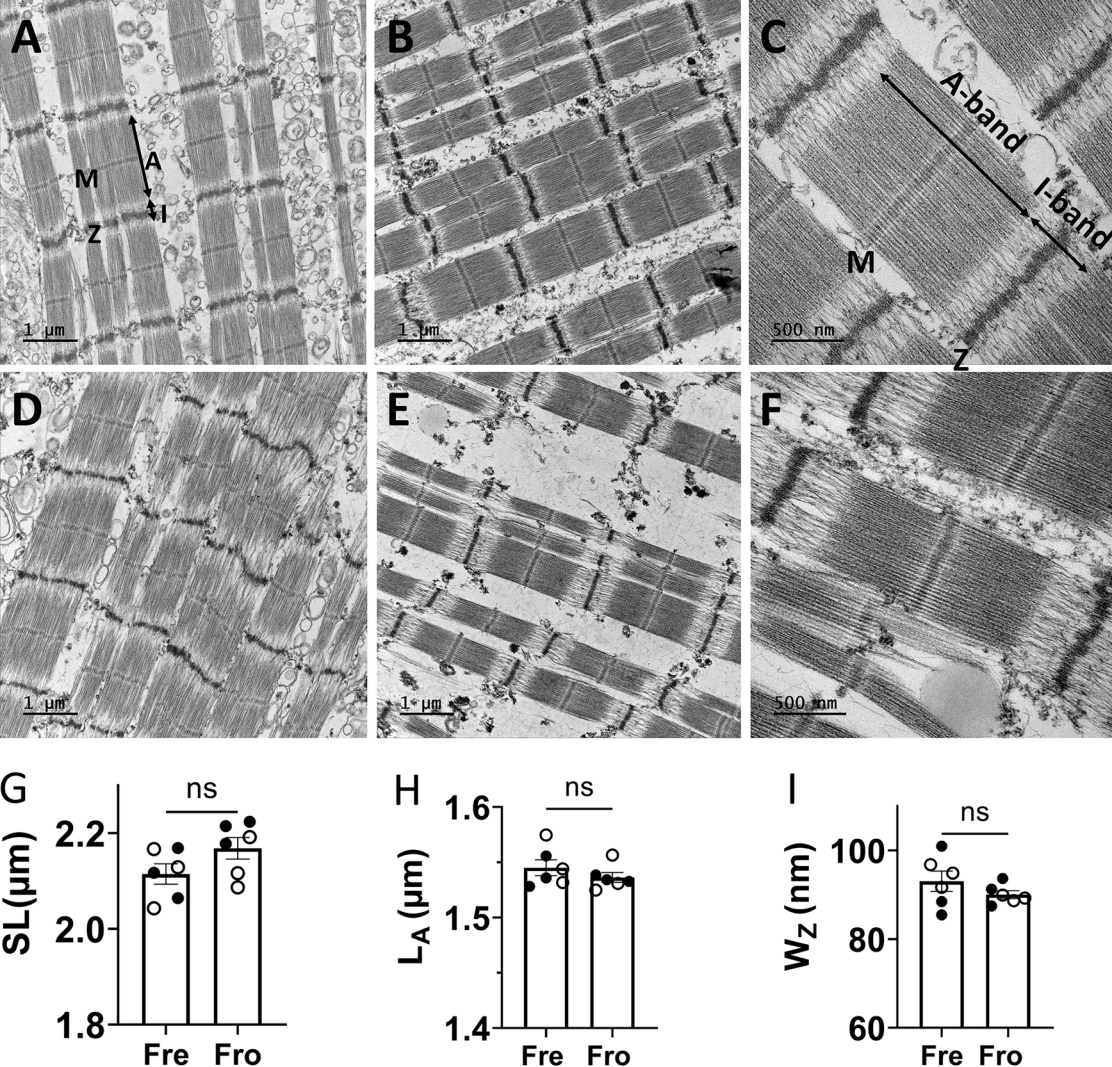
**Electron micrographs of fresh and frozen myocardium. (A–F)** Representative electron microscope images of fresh (A–C) and frozen (D–F) porcine myocardium in the longitudinal section at low and high magnification. **(G–I)** Sarcomere length (SL; G), A-band length (H), and Z-line width (I) are shown for fresh (Fre) and frozen (Fro) tissue. ns: P *≥* 0.05. Solid and open symbols are from different biological repeats. The error bars in the graphs are SEM.

Statistical analyses were performed using GraphPad Prism 9 (Graphpad Software). The results are given as mean ± SEM unless otherwise stated. Two-tailed nested *t* tests were used for the data in Figs. 1, 2, and 3. Force calcium relationships were fit to a Hill curve to estimate parameters: maximal calcium-activated force (F_max_), calcium sensitivity (EC_50_), and Hill Coefficient. Symbols in figures: ns: P *≥* 0.05, and ****: P < 0.0001.

## Results

### Myofilament lattice in fresh and frozen porcine myocardium

X-ray diffraction patterns (Fig. 1) from permeabilized fresh and frozen porcine myocardium showed comparable features (Fig. 1, A and B). Qualitatively, the equatorial reflections arising from the hexagonally packed thick and thin filaments (Ma and Irving, 2022) are strong and sharp in diffraction patterns from both fresh and frozen. The equatorial intensity ratio, which reports the disposition of myosin heads relative to actin-containing thin filaments (Ma and Irving, 2022), is not significantly different between the freshly (0.25 ± 0.01, *n* = 18) and previously frozen (0.23 ± 0.01, *n* = 21; P = 0.13) samples (Fig. 1 C). The lattice spacing (d_1,0_), which is proportional to the inter-thick filament distance, however, is significantly larger (by ∼ 2%) in previously frozen tissue (40.96 ± 0.01 nm, *n* = 21) than freshly permeabilized tissue (40.11 ± 0.15 nm, *n* = 18; P < 0.001; Fig. 1 D).

To further investigate the lattice arrangement, we visualized cross-sectional views of sarcomere A band from chemically fixed fresh and frozen porcine myocardium by thin-section EM. We selected regions where both the thick and thin filaments were present and found that six thin filaments (red hexagons) formed a regular hexagonal lattice with one thick filament in the middle (yellow circles) in micrographs of both fresh (Fig. 1 E) and frozen (Fig. 1 F) porcine myocardium. The regularity of the lattice was similar in the fresh and frozen preparations. To quantify the lattice ordering, we extracted the peak width parameters, σ_d_ and σ_s,_ from the equatorial x-ray reflections (Ma et al., 2018a). σ_d_ is proportional to the amount of heterogeneity in interfilament spacing (Δd10/d10) among the myofibrils, and σ_s_ is proportional to the degree of paracrystalline (liquid-like) disorder of the myofilaments in the hexagonal lattice (Yu et al., 1985). There was no significant difference in σ_d_ (1.25 ± 0.07 vs. 1.31 ± 0.05, P = 0.78, Fig. 1 G) and σ_s_ (0.11 ± 0.019 vs. 0.07 ± 0.015, P = 0.26, Fig. 1 H) between fresh and frozen myocardium. Together, these data from EM and x-ray diffraction indicate the myofilament lattice arrangements were well preserved by cryopreservation as compared with freshly prepared myocardium samples.

### Meridional x-ray reflections from fresh and frozen porcine myocardium

The spacing of the M3 reflection (S_M3_), whose intensity is dominated by contributions from the myosin heads, reports the axial periodicity of the heads, while the spacing of the M6 reflection (S_M6_), whose intensity is dominated by contributions from the thick filament backbone, reports periodicities within the thick filament backbone (Linari et al., 2000; Huxley, 2004; Reconditi, 2006; Fig. 2). There are no significant differences in S_M3_ (fresh: 14.35 ± 0.004 nm, *n* = 18; frozen: 14.36 ± 0.003 nm, *n* = 18; P = 0.38) and S_M6_ (fresh: 7.18 ± 0.003 nm, *n* = 18; frozen: 7.19 nm ± 0.003, *n* = 18; P = 0.25) between fresh and frozen porcine myocardium (Fig. 2, A and B). In the resting state, the large majority of the myosin heads are quasi-helically ordered on the surface of the thick filament and give rise to the characteristic myosin-based layer lines so that the I_MLL1_ (430 Å repeat) is an indicator of the degree of ordering of the myosin heads. There are no significant differences in I_MLL1_ between the fresh (0.87 ± 0.06, *n* = 13) and frozen (0.73 ± 0.04, *n* = 17; P = 0.23) porcine myocardium (Fig. 2 C). The intensity of M3 (I_M3_) is another indicator of the ordering of the myosin heads, and there is no significant difference in I_M3_ between the fresh (0.57 ± 0.02, *n* = 17) and frozen porcine (0.55 ± 0.03, *n* = 18; P = 0.80) myocardium (Fig. 2 D). These results from x-ray diffraction showed that the structural integrity of myofilaments in porcine myocardium at the resolution of the x-ray patterns is largely retained in liquid nitrogen–frozen, −80°C–stored, and then thawed tissue.

### Electron micrographs of sarcomeres in longitudinal sections

We also obtained longitudinal sections of the sarcomeres by EM (Fig. 3). Qualitatively, the freshly permeabilized porcine myocardium showed excellent EM ultrastructure, with well-defined A-bands, I-bands, M-lines, and Z-lines constituting the sarcomeres; filament organization in the frozen tissue was also good, but not as well defined or consistent in the samples we examined. The lengths of the A bands were the same between the fresh (1.55 ± 0.007 μm, *n* = 6) and frozen porcine (1.54 ± 0.004 μm, *n* = 6; P = 0.32) myocardium. However, the edge of the A-band (red lines, Fig. 3, A–F) is better defined in micrographs from fresh samples. The sarcomere lengths from frozen samples (2.17 ± 0.02 μm) were slightly, but not significantly, larger than from the fresh samples (2.12 ± 0.02 μm, P = 0.28; Fig. 3 G) but both were well within the physiological sarcomere length range. It is, therefore, unlikely that the less sharp A-band edges in frozen samples were caused by over-stretching during the sample preparation. The width of the Z-line is essentially the same in the fresh (93.06 ± 1.14 nm, *n* = 6) and frozen (90.06 ± 0.93 nm, *n* = 6; P = 0.25) myocardium. The Z-lines appeared to be approximately straight in the freshly permeabilized samples, but more irregular in micrographs from previously frozen tissue (Fig. 3, A–F).

### Contractility of fresh and frozen porcine myocardium

**Figure 4.**
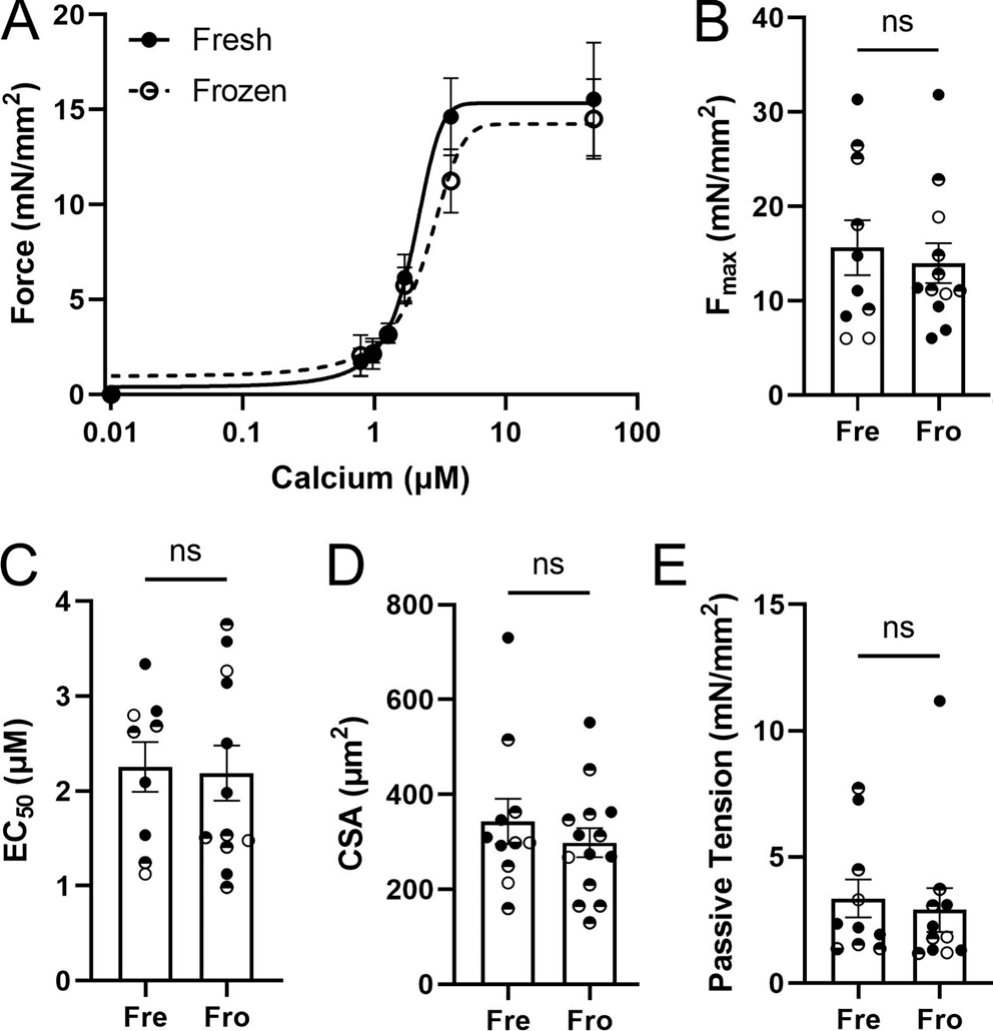
**Contractile properties from fresh and frozen porcine myocardium. (A)** Skinned myocyte force–calcium relationships from porcine left ventricular myocardium either isolated immediately after sacrificing the animal or flash-frozen and subsequently isolated. Both fresh and frozen cells were isolated from the same three pigs from tissue samples immediately adjacent to each other. **(B and C)** Summary F_max_ (B) and EC_50_ (C) data corresponding to the force–calcium graph in A. **(D)** Cross-sectional area (CSA) was calculated from each cell assuming an elliptical cross-section, with no difference between the groups. **(E)** Passive tension at sarcomere length of 2.1 μm was also not different between the fresh and previously frozen myocytes. Solid, open, and half open/closed symbols are from different biological repeats. ns: P *≥* 0.05. The error bars in the graphs are SEM.

To compare the contractility of fresh and frozen porcine myocardium, we assessed force–calcium relationships in isolated permeabilized cardiomyocytes (Fig. 4). Permeabilized porcine myocyte preparations showed a classic sigmoidal force vs. calcium concentration relationship (Fig. 4 A). There was no significant difference in normalized maximally activated force (F_max_) between fresh (15.6 ± 2.9 mN/mm^2^, *n* = 10) and frozen (14.00 ± 2.1 mN/mm^2^, *n* = 12; P = 0.65, Fig. 4 B) tissues. Similarly, there was no significant difference in calcium sensitivity between the frozen tissue (2.19 ± 0.29 μM, *n* = 12) compared with fresh (2.25 ± 0.26 μM, *n* = 10, P = 0.87, Fig. 4 C). Force values were calculated by normalizing raw force by the myocyte cross-sectional area that was also not different between the groups. Lastly, passive tension at a sarcomere length of 2.1 μm (the sarcomere length at which the data in Fig. 4 A was collected) was also not different between the groups (fresh = 3.4 ± 0.7 mN/mm^2^, frozen = 2.9 ± 0.8 mN/mm^2^, P = 0.70).

## Discussion

Cryopreservation of ultrastructural features of muscle tissue at high resolution is a challenging task. Special devices and ultrarapid freezing, thawing, and fixation procedures have been developed to better preserve muscle ultrastructural features (Padron et al., 1988; Craig et al., 1992). Ochala et al. (2010) reported that high-quality x-ray diffraction patterns can be acquired from liquid nitrogen–preserved human skeletal muscle biopsy samples after an elaborate prefreezing treatment and in the presence of sucrose as a cryoprotectant. Here, we preserved the porcine myocardium by simply immersing chunks of muscle tissue (∼0.5 cm^3^) in liquid nitrogen without any pretreatment, seemingly a slow and crude way of preserving tissue, although it is a widely used technique for freezing purified proteins. Similarly, clinical specimens are often preserved in this way and it has been shown that ultrastructural features of such samples can be well conserved when thawed and fixed in glutaraldehyde (Galhuber et al., 2021), consistent with our findings. Strikingly, when flash-freezing (by impact with liquid helium–cooled copper block) is used to ultrarapidly freeze (<10 ms) striated muscle tissue, followed by freeze substitution to chemically preserve frozen components, molecular structural preservation is superb within 5–10 µm of the surface (Craig et al., 1992; Luther et al., 2011) but deteriorates rapidly at deeper levels (>10 µm), with severe ice crystal damage apparent (as distortion of the filament lattice; Padron et al., 1988). Liquid nitrogen freezing, which is orders of magnitude slower, would be expected to have much worse consequences, with formation of large ice crystals that damage and distort structure. Why such deleterious effects are not seen with our cruder methodology is not clear. The damage seen in ultrarapidly frozen, freeze-substituted tissue (Padron et al., 1988) might be visible because freeze substitution with osmium chemically fixes the specimen while still frozen, preserving the damage, while thawing first and then fixing allows it to reverse. We show that at the resolution of small-angle x-ray diffraction patterns (at best 5 nm), molecular structure is largely retained, with minimal changes in the spacings, intensities, and sharpness of major diffraction peaks caused by freezing. In the current study, we only systematically investigated the effectiveness of cryopreservation at the 1-mo time point. We have reported excellent x-ray diffraction patterns from previously frozen porcine myocardium with various storage durations up to 4 yr (Ma et al., 2023).

The only significant change was a small (∼0.8 nm) expansion of the interfilament lattice spacing (d_1,0_) in the previously frozen specimens. A 0.8-nm difference in d_1,0_ in skinned muscle, where the stabilizing forces determining d_1,0_ are very weak, could be for a variety of reasons (Millman, 1998) and hard to pin down. We can only speculate what these reasons might be. Small differences in the degree of membrane disruption might be expected to result in detectable differences in d_1,0_. In fresh muscle, membrane disruption is due to Triton alone. While the degree of disruption of the sarcolemma allowing ingress of calcium is clearly similar in fresh and previously frozen tissue, since response to calcium is not different, there may be subtle differences in the degree of disruption of other internal membranes in the two preparations. Liquid nitrogen freezing, as we have done, will result in significant disruption of all cellular membranes, and possibly the extracellular matrix (Padron et al., 1988), in addition to the additional membrane disruption provided by Triton skinning, possibly allowing additional lattice expansion by reducing compressive forces on the lattice. Titin-based passive tension, which can be substantial at longer sarcomere length, can result in large changes in d_1,0_ (Fukuda et al., 2005; Hessel et al., 2022) so that small differences in preservation of titin in fresh and frozen tissues might be expected to result in detectable changes in d_1,0_. While this possibility cannot be excluded, we believe the lattice spacing difference is unlikely to be due to titin degradation because (1) there are no significant differences in passive tension at sarcomere length 2.1 (Fig. 4 E); (2) and the S_M6_ values are about the same at sarcomere length 2.0 (Fig. 2 B) in fresh and previously frozen muscle, suggesting that titin is not significantly altered by the freezing process. It is worth noting that the d_1,0_ in permeabilized porcine myocardium (∼40 nm) is substantially smaller than in skinned rodent myocardium (∼42 nm; Irving et al., 2000; Powers et al., 2019; Yuan et al., 2022) at similar sarcomere lengths. We can only speculate at this point on the reasons for smaller d_1,0_ in permeabilized porcine myocardium than in rodent myocardium, but they could result from the differences in sarcomeric protein isoforms such as myosin heavy chain (α vs. β; Walklate et al., 2016) and titin (Cazorla et al., 2000).

Force–calcium relationships are commonly assessed in cardiomyocytes isolated from previously frozen tissue, even for rodent studies. A previous study compared the effect of flash-freezing on force–calcium relationships in human myocardium and observed no effect on maximal force generation but a modest decrease in calcium sensitivity (Milburn et al., 2022). The data presented here in porcine myocardium mostly recapitulated this study in humans, showing no effect on maximal force generation or calcium sensitivity (EC_50_). The modest change in calcium sensitivity in human study (Milburn et al., 2022) and the slight difference in d_1,0_ presented here, however, does underscore the importance of not mixing fresh and frozen tissues in any given study.

Myofilaments isolated from liquid nitrogen–frozen and then thawed striated muscles appear to be very well preserved at the molecular level (Al-Khayat et al., 2013; Kensler et al., 2017), consistent with preservation of the myosin layer lines and overall EM ultrastructure in the current study. It appears that any ice crystal damage that does occur to myofilaments during the relatively slow freezing used in our study may be reversed during the thawing process, leading to final good preservation. By allowing storage of large amounts of material from individual hearts, this procedure can lead to more consistent experiment results for many studies by eliminating potential animal-to-animal variability but can also lead to reducing the number of animals needed by eliminating the need for a constant supply of fresh tissue. Additionally, the majority of human myocardium studies are performed on previously frozen heart muscles. The current comprehensive structural and functional study validates this preparation as an excellent test bed for biophysical studies of cardiac muscle.

## Data Availability

The datasets generated or analyzed during this study are included in this article. The raw data are available from the corresponding authors (W. Ma: wma6@iit.edu; T. Irving: irving@iit.edu) upon reasonable request.
